# Upper limb prostheses: a narrative review of hand, wrist, and elbow devices

**DOI:** 10.3389/fresc.2026.1845424

**Published:** 2026-07-15

**Authors:** Alessio Fricano, Mattia Giuseppe Viva, Mario Vetrano, Marco Paoloni, Giuseppe Giannicola, Francesco Agostini, Giovanni Galeoto, Sveva Maria Nusca, Francescaroberta Panuccio, Irene Gennarelli, Massimiliano Murgia, Massimiliano Mangone

**Affiliations:** 1 Department of Anatomy, Histology, Forensic Medicine and Orthopedics, Sapienza University of Rome, Rome, Italy; 2 Department of Experimental Medicine, University of Salento, Lecce, Italy; 3 Physical Medicine and Rehabilitation Unit, Sant'Andrea Hospital, Sapienza University of Rome, Rome, Italy; 4 Department of Human Neurosciences, Sapienza University of Rome, Rome, Italy; 5 Department of Mathematics, Computer Science and Physics, University of Udine, Udine, Italy; 6 Rehabilitation Unit, Order of Malta, San Giovanni Battista Hospital, Rome, Italy

**Keywords:** myoelectric control, prostheses and implants, prosthetic elbow joint, prosthetic hand, prosthetic wrist, upper limb amputation

## Abstract

**Background:**

Upper limb amputation is among the most functionally disabling conditions, affecting an estimated 1.6 million people in the United States, with prevalence projected to double by 2050. Despite decades of technological progress, device abandonment rates remain persistently high, up to 45% for body-powered and 35% for myoelectric prostheses in the paediatric population, due to inadequate functionality, poor sensory feedback, discomfort, and social stigma.

**Objectives:**

This narrative review aims to provide a structured and up-to-date synthesis of upper limb prosthetic technologies, with a focus on hand, wrist, and elbow devices, analysing their structural characteristics, control mechanisms, clinical evidence on functional outcomes, and user satisfaction.

**Methods:**

A structured literature search was conducted across PubMed, Scopus, Web of Science, and the Cochrane Library, combining terms related to upper limb amputation and prosthetic devices. A primary corpus of peer-reviewed articles was selected to cover the full spectrum of upper limb prosthetics, from foundational biomechanics to advanced bionic and neural interface systems; a narrative approach was adopted to accommodate the heterogeneity of available study designs.

**Results and conclusions:**

Across all device categories, no single prosthetic solution proved universally superior. Each category presents distinct functional, biomechanical, and psychosocial trade-offs, with prescription choices driven by amputation level, residual musculature, user priorities, and activity demands. Two limitations recur across every category. The first is the instability of surface EMG signals, which constrains reliable myoelectric control; the second is the absence of effective sensory feedback, which raises cognitive load and contributes to abandonment. Bridging the gap between laboratory performance and real-world usability, through more robust control algorithms, meaningful sensory restoration, and a shift toward user-centred, outcomes-driven development, remains the central challenge for the future of upper limb prosthetics.

## 1 Introduction

Amputation of an upper limb is one of the most functionally disabling injuries a person can suffer, putting severe restrictions on their ability to perform daily living activities (ADLs), be productive at work, and participate in social life, with a significant psychological impact.

**Table 1:** Upper limb prostheses: comparative overview.

Type	Category	Description	Advantages	Disadvantages	Common use
HAND PROSTHESES					
Passive/Cosmetic Hand	Passive	Silicone or thermoplastic device with no internal mechanism; may allow manual finger repositioning.	Lightweight, low-maintenance, water-resistant; restores body image and psychosocial well-being; highest prevalence among UL devices (∼1 in 3 users).	No active function; limited grip capability; primarily aesthetic.	Congenital limb loss; users prioritising appearance and social integration over function.
Body-Powered (BP) Terminal Device/Hook	Body-Powered	Cable-and-harness system translating shoulder/residual limb movement into VC or VO terminal device actuation.	Durable, lightweight, reliable in harsh environments (humidity, dust); provides proprioceptive feedback via cable forces; lower cost and maintenance.	Unaesthetic (hook); requires wide shoulder range of motion and adequate residual limb length; harness may cause discomfort.	Physically demanding or outdoor work; users seeking durability and sensory feedback.
Single-DOF Myoelectric Hand	Externally Powered	Dual-site sEMG control driving one DC motor to simultaneously open/close all fingers.	More natural appearance than hook; proportional force/speed control; established rehabilitation pathways; moderate cost.	Single grip pattern requires sequential mode-switching (cognitively demanding); signal instability due to sweat, electrode shift, or fatigue.	Transradial amputees needing functional everyday use with moderate complexity.
Multi-Articulating Myoelectric Hand	Externally Powered	Independently motorised fingers enabling multiple grip configurations (pinch, key, power, etc.).	Multiple grip patterns; improved long-term user satisfaction and quality of life (≥6 months); greater dexterity for ADLs.	Heavier and more expensive; grip-mode switching remains slow and unintuitive; no statistically significant gain in SHAP/BBT over single-DOF.	Users requiring versatile grasps and willing to invest time in training.
3D-Printed Prosthetic Hand	Passive/Functional	Additively manufactured devices; mainly body-powered or passive; highly customisable.	Very low material cost (≤USD 500); rapid customisation (colour, size, shape); accessible in low-resource settings; suitable for children.	Limited durability and fine motor precision; inconsistent quality control; not yet widely adopted in clinical practice.	Paediatric patients; amputees in developing countries lacking prosthetic care.
WRIST PROSTHESES					
Passive Wrist Unit	Passive	Allows manual repositioning of the wrist using the opposite hand; no power source required.	Simple, lightweight, low-cost; improves overall prosthesis functionality; users report greater satisfaction vs. fixed wrist.	Requires use of the contralateral hand to reposition; no active movement during tasks.	Users who need occasional wrist pre-positioning without active control.
Powered Wrist Rotator	Externally Powered	Electric motors (EMG- or IMU-controlled) enabling active pronation/supination and/or flexion/extension.	Restores active wrist movement; reduces compensatory trunk/shoulder movements; improves hand pre-positioning for grasping.	Heavier and more complex; higher cost; some tasks (e.g., cup-holding) may be harder than with BP; EMG channel competition with hand.	Transradial users who need dynamic wrist positioning; integration with multi-DOF systems.
ELBOW PROSTHESES					
Body-Powered (BP) Elbow Unit	Body-Powered	Cable-and-harness system using trunk and shoulder movement to flex/extend the prosthetic elbow.	Lightweight, durable, reliable; provides proprioceptive feedback via cable; lower cost and maintenance.	Requires adequate residual limb length and shoulder mobility; cannot simultaneously operate elbow and terminal device without complex switching; harness discomfort.	Transhumeral amputees with good shoulder mobility; active/outdoor users.
Myoelectric Elbow Unit	Externally Powered	EMG-controlled electric motor for elbow flexion/extension; no harness required.	More natural appearance (no visible straps); better suited for proximal amputees with limited shoulder mobility; compatible with TMR.	Sequential control of elbow + wrist + hand is cognitively demanding; slower and less intuitive; heavier and costlier.	Proximal transhumeral or shoulder-disarticulation patients; users who prioritise aesthetics.
Hybrid Elbow–Hand System	Hybrid	BP elbow combined with a myoelectric terminal device (hand or hook) and often a myoelectric wrist.	Simultaneous control of elbow (BP) and hand (myoelectric); lighter than fully electric; greater grip strength than fully BP; pragmatic for complex amputations.	Harness discomfort (especially short transhumeral); straps may be aesthetically unappealing; fitting complexity increases.	Proximal transhumeral and shoulder-disarticulation amputees; patients for whom full myoelectric control is too complex.

ADL, activities of daily living; BP, body-powered; DOF, degree of freedom; EMG, electromyography; IMU, inertial measurement unit; SHAP, Southampton hand assessment procedure; BBT, box and block test; sEMG, surface EMG; TMR, targeted muscle reinnervation; UL, upper limb; VC, voluntary closing; VO, voluntary opening.

In 2005 alone, approximately 1.6 million people in the United States were living with a limb loss, of whom 41,000 had undergone major upper limb amputations, excluding finger amputations (1). Demographic projections suggest that this prevalence could double by 2050, due in part to the diabetes epidemic and the ageing population (2).

Epidemiological data on the prevalence and incidence of upper limb amputations vary considerably from one country to another, and many studies have not distinguished between upper and lower limb amputations, thereby limiting the accuracy of global comparisons and the development of targeted policies (3). The main cause of amputation varies according to the socio-economic context; peripheral vascular disease and diabetes have become the leading causes of amputation in developed countries, whereas in developing countries, trauma remains the main cause of amputation (4). The military context further highlights this finding: data on US veterans indicate that transradial and transhumeral amputations are the two most common levels of amputation, with transradial amputation (36.1%) in first place, followed by transhumeral amputation (30.4%) (5). Throughout this review the terms transradial (below-elbow) and transhumeral (above-elbow) are used; the parenthetical glosses are given here at first occurrence and the standardised terms are used thereafter.

The primary aim of upper limb prosthetic rehabilitation is to restore meaningful function, reduce disability and promote psychosocial well-being following amputation or congenital absence of a limb. Over the last century, prosthetic technology has advanced. Despite this notable technological evolution, device non-use remains persistently high. In a review assessing the abandonment rate of upper limb prostheses, average rejection rates of 45% and 35% were found for body-powered and electric prostheses respectively in the paediatric population. In the adult population, significantly lower rejection rates were observed for both types of devices (26%) and for electric prostheses (23%) (6). The most commonly cited reasons for non-use include inadequate functionality, excessive device weight, lack of sensory feedback, discomfort at the residual limb–sock interface, and social stigma (7).

These persistent shortcomings highlight the need for a comprehensive and up-to-date synthesis of the scientific evidence behind current prosthetic technologies. This narrative review aims to provide a structured overview of upper limb prostheses, organised by amputation level and prosthetic category, with a particular focus on hand, wrist and elbow prosthetics. Following an introduction to classification systems and considerations regarding amputation level, the review describes the structural characteristics and control mechanisms of each class of device, summarises the clinical evidence on functional outcomes and user satisfaction, and discusses the main differences, limitations and future prospects of the field.

## 2 Background

### 2.1 Levels of upper limb amputation

Amputation can be classified according to the following levels, from distal to proximal: partial hand amputation (8), wrist disarticulation, transradial amputation, elbow disarticulation, transhumeral amputation, shoulder disarticulation and forequarter amputation (9).

The level of amputation determines the residual musculature available for prosthetic control. Transradial amputees retain the biceps, triceps and, depending on the length of the residual limb, the forearm pronators and supinators, preserving sufficient musculature to allow forearm rotation as a linear function of the residual forearm length (10). Transhumeral amputees typically retain only the biceps and triceps as primary signal sources, unless nerve transfer surgical procedures are employed (11). At the level of shoulder or anterior quarter disarticulation, no accessible muscles of the forearm or arm remain, necessitating alternative control interfaces such as targeted muscle reinnervation (TMR) (12) or osseointegrated neural electrodes (13).

### 2.2 General classification of upper limb prostheses

Upper limb prostheses are broadly divided into passive and active categories (9). Passive prostheses can also be designed to look more natural; however, their primary purpose is to protect sensitive areas of the residual limb and to restore the limb's length, thereby improving the functional use of the affected side. Their natural appearance also helps to restore the individual's body image, contributing to their psychological well-being.

Active prostheses generate motion through one of two mechanisms. Body-powered (BP) systems transmit cable forces via a shoulder harness, translating, proximal body movements into distal terminal device actuation. They are generally lighter, more durable, more resistant to environmental conditions (such as humidity and dust), provide secondary proprioceptive feedback to the user, have a lower initial price, and incur lower maintenance costs than electrically powered options.

Externally powered systems are most commonly myoelectric: electromyographic (EMG) signals from residual stump muscles are detected by surface electrodes embedded in the prosthetic socket and converted by a microprocessor to drive battery-powered electric motors. Although they have higher production costs, are heavier and require more frequent repairs, they offer greater precision in gripping force.

Hybrid devices combine both paradigms, typically a body-powered elbow with a myoelectric terminal device, and are most commonly employed at the transhumeral level. In general, the main advantages of hybrid devices are that they allow for the simultaneous control of multiple components; they are lighter than a fully electric prosthesis and they offer greater grip strength than a prosthesis operated entirely by the body.

### 2.3 Clinical implications of amputation level for prosthetic prescription

The level of upper limb amputation has profound implications for prosthetic prescription, as it directly influences both the biomechanical and control-related characteristics of the prosthetic system. As the amputation level becomes more proximal, the number of available anatomical segments and residual muscles decreases, leading to a progressive reduction in independent control sites for myoelectric signal acquisition. This limitation significantly affects the ability to generate distinct and reliable control inputs, thereby increasing the complexity of device operation and often requiring more sophisticated control strategies or hybrid solutions (1, 11).

In transradial amputations, users typically retain sufficient residual musculature to allow relatively intuitive control of terminal devices, especially in body-powered or conventional myoelectric systems. In contrast, transhumeral amputations require the coordinated control of multiple joints, including the elbow, wrist, and hand, often with a reduced number of available control signals. This mismatch between the number of controllable degrees of freedom and the available control inputs frequently necessitates sequential control schemes or mode switching, which can increase cognitive load and reduce usability during complex functional tasks (14).

More proximal amputations also entail greater reliance on compensatory trunk and shoulder movements, particularly during reaching and manipulation. These strategies stem from two sources: the mechanical limitations of the prosthesis and the constraints of its control. Over time, they may reduce movement efficiency and compromise musculoskeletal health (15, 16). As a result, prosthetic prescription at higher amputation levels often involves a trade-off between functional capability and control complexity.

Overall, the level of amputation represents a key determinant in selecting the most appropriate prosthetic configuration, influencing not only the type of components used but also the control strategy, training requirements, and expected functional outcomes. A comprehensive understanding of these factors is essential for optimizing prosthetic design and improving user acceptance in clinical practice (6, 17).

## 3 Methods

This narrative review was conducted in accordance with established methodological frameworks for narrative reviews (18) and reported in line with the Scale for the Assessment of Narrative Review Articles (SANRA) (19).

A structured literature search was performed across four electronic databases: PubMed, Scopus, Web of Science, and the Cochrane Library. The search combined controlled and free-text terms with Boolean operators, as follows: (“amputee*” OR “amputation” OR “limb loss” OR “upper limb amput*” OR “transradial amput*” OR “transhumeral amput*” OR “wrist disarticulation” OR “elbow disarticulation”) AND (“upper limb prosthes*” OR “upper extremity prosthes*” OR “prosthetic hand” OR “hand prosthes*” OR “wrist prosthes*” OR “elbow prosthes*” OR “myoelectric prosthes*” OR “body-powered prosthes*”).

No date restriction was applied, so as to capture both the foundational literature and the most recent technological developments in a field characterised by a comparatively small and dispersed evidence base. The search was restricted to peer-reviewed articles published in English. Grey literature (e.g., conference abstracts without full peer review, dissertations, and manufacturer white papers) was not searched systematically; however, the reference lists of all included articles and of relevant existing reviews were hand-searched to identify additional sources not retrieved by the database queries.

Eligibility criteria: Articles were eligible if they addressed the structure, control mechanisms, functional outcomes, or user-reported outcomes of hand, wrist, or elbow prosthetic devices for upper limb amputation or congenital limb absence. Both primary studies (across all designs, given the heterogeneity of the field) and secondary syntheses were eligible.

Exclusion criteria: Studies were excluded if they (i) addressed surgical technique without any prosthetic-device-related outcome (e.g., purely oncological case reports); (ii) concerned exclusively lower limb prosthetics; (iii) were not available in English; or (iv) did not provide retrievable peer-reviewed content. The review focused on adult upper limb amputees; paediatric-specific evidence was cited where directly relevant but did not constitute the primary focus.

A predefined corpus of peer-reviewed articles, identified by their Digital Object Identifiers (DOIs), constituted the primary source base. These articles were selected to span the full scope of upper limb prosthetics, from foundational anatomy and biomechanics to state-of-the-art bionic systems and neural interfaces, and the final selection was guided by relevance in representing key technological developments, clinical applications, and emerging trends. Consistent with its narrative design, this review did not apply a formal risk-of-bias assessment and did not undertake quantitative synthesis; the rationale and implications of this choice are addressed explicitly in the Limitations (Section 6).

## 4 State of the art in upper limb prosthetic devices

### 4.1 Prosthetic hand devices

#### 4.1.1 Passive and cosmetic prosthetic hands

Passive prostheses are one of the oldest and most widespread categories in the field of upper limb prosthetics. They are divided into two main subgroups: cosmetic prostheses, whose primary purpose is to restore the aesthetic appearance of the lost limb, and passive prosthetic tools, designed to support simple tasks such as pushing, pulling and carrying objects. Although they receive little attention in the scientific literature compared to their active alternatives, passive prostheses are used by approximately one in three amputees, giving them the highest prevalence among upper limb prosthetic devices (20).

Several cross-sectional studies have found that users of cosmetic prostheses report quality-of-life scores comparable to or even higher than those of myoelectric users, underscoring the psychosocial value of body image restoration independent of functional gain (21).

Patients with congenital limb loss tend to prefer aesthetic hand prostheses over other types of prostheses for aesthetic reasons, since social integration is the primary consideration rather than the functionality provided by the prosthesis (22). From a technical perspective, static passive prostheses are made from silicone or thermoplastic materials, with particular attention given to matching the colour of the patient's skin. Adjustable versions allow for manual repositioning of the fingers to facilitate some basic grips, without any internal actuating mechanism (20). They contain no mechanical or electronic moving parts, making them lightweight, low-maintenance, and resistant to water and dirt.

#### 4.1.2 Body-powered terminal devices and functional hands

Body-powered (BP) prostheses are one of the most established functional solutions in prosthetic rehabilitation. The operating principle is based on the transmission of movement, typically from the contralateral shoulder or the residual limb, via a Bowden cable connected to a harness that controls the opening or closing of the terminal device (hook) (23).

Terminal devices are divided into two main types: voluntary closing (VC) and voluntary opening (VO), both with their own specific biomechanical and functional characteristics. Comparative studies between VC and VO modes indicate that users perform significantly better when they can switch between the two modes depending on the task at hand, with scores on the Southampton Hand Assessment Procedure (SHAP) averaging 4–7 points higher than when using only one mode (24).

Although the hook does not have any aesthetic appeal, it is historically regarded as superior to the artificial hand in terms of practical functionality, mechanical strength and versatility for physically demanding activities (25).

A further advantage of BP prostheses is the ability to use partial proprioceptive feedback through the forces transmitted to the brace, providing the user with indirect information about the grip force applied by the terminal device (26).

#### 4.1.3 Single degree-of-freedom (DOF) myoelectric hands

Conventional myoelectric hands operate via dual-site electrode control: contraction of one muscle group (typically wrist flexors) closes the hand while the antagonist (wrist extensors) opens it (27). Surface electrodes embedded in the prosthetic socket detect surface EMG (sEMG) signals, which are amplified and processed by an onboard microcontroller to drive a single DC motor coupled to a tendon or gear mechanism that actuates all fingers simultaneously.

The EMG signal has been used to control prosthetic hands since 1948. The first commercial production of prosthetic hands using this technology began in 1957 at the Central Research Institute of Prosthetics in Moscow (28).

Proportional control, where EMG amplitude governs both grip speed and force, enables a degree of force modulation but is sensitive to electrode displacement, perspiration-induced impedance changes, and muscle fatigue (29).

The primary limitation of single-DOF systems is that multiple grip patterns must be sequentially selected using co-contraction or EMG switching, a cognitively demanding process that slows task execution (30). Nevertheless, their relative simplicity, moderate cost, and established rehabilitation pathways make them the dominant clinical prescription for transradial amputees worldwide.

#### 4.1.4 Multi-articulating myoelectric hands

The advent of multi-articulated myoelectric hands has marked a revolution in upper limb prosthetics, offering for the first time the ability to perform a variety of grips (pointing index finger, pinch grip, key grip) using a set of independently motorised fingers. Belter et al. conducted a systematic review of the mechanical characteristics of these devices, highlighting significant differences in terms of weight, gripping force and the number of available gripping configurations (31).

Despite their undoubted mechanical superiority over single-DOF hands, clinical data suggest that the functional benefits of multi-jointed hands remain partially unfulfilled under conventional myoelectric control: switching between grip modes is slow and unintuitive, limiting the spontaneity of use (32).

Clinical evidence on multi-articulating hands has yielded mixed results. A systematic evaluation and multiple cross-sectional studies found no statistically significant difference in SHAP or Box and Block Test (BBT) scores between multi-grip and single-grip myoelectric prostheses in transradial amputees (33).

However, it has been shown that, over follow-up periods of six months or more, multi-grip devices lead to improved user satisfaction, a perceived reduction in the difficulty of performing activities, and an improved quality of life compared with single-grip alternatives (34).

#### 4.1.5 3D-printed prosthetic hands

3D printing has opened up a radically new approach to the design and distribution of prosthetic hands, breaking down financial barriers and increasing the scope for customisation. According to a systematic review conducted by Ten Kate et al., 58 different models of 3D-printed prosthetic hands were already available at the time of publication, the majority of which were intended for paediatric patients, with a maximum material cost of around USD 500. Contrary to what is often claimed, 3D printing is not necessarily cheap, although it does offer interesting possibilities for customisation in terms of design, colour, shape and size without the need to modify the production machinery (35).

A later systematic review of 3D-printed prostheses in developing countries highlighted that the low cost of materials, the possibility of customising the design, and the accessibility of the technology make these devices a solution with great potential for amputees in developing countries who lack adequate prosthetic care (36).

On the other side, although they improve gross motor function and participation in daily activities, their limited durability and fine motor precision remain obstacles to their widespread clinical adoption (37).

Beyond cost and customisation, the maturity of 3D-printed devices is constrained by a set of challenges that have not yet been systematically resolved: durability, sufficient grip strength, reproducibility across prints, and broad user acceptance (38). Functional designs for multi-articulated hands and sockets are freely available and already used by patients at home, but the same openness that drives accessibility complicates standardisation and quality control (38). A recent systematic review of clinical outcomes confirms that, although these devices improve gross motor function and participation, limitations in durability and fine motor precision continue to restrict their wider clinical adoption (37).

### 4.2 Prosthetic wrist units

Wrist prosthetic units are a fundamental component of upper limb prostheses, as the natural wrist plays a decisive role in positioning the hand in space to enable the performance of activities of daily living. Biomechanical studies have demonstrated that a specific range of wrist motion is required to perform common tasks such as reaching, grasping, and object manipulation, including flexion–extension and pronation–supination movements that allow proper alignment of the terminal device with the target object (39, 40). Pronation/supination is the degree of freedom most frequently requested by upper limb prosthesis users, followed by flexion/extension and radial–ulnar deviation (41). Despite significant advances in the field of multi-jointed prosthetic hands, wrist units have historically received less attention in research, despite having a significant impact on the overall functionality of the device (20). The prosthetic wrist allows the hand to be pre-positioned before performing a task, preventing the onset of unwanted compensatory movements involving the trunk and shoulders, which can lead over time to joint overload injuries (41). The integration of a wrist rotation module into a prosthetic hand for partial hand amputees has been shown to restore normal upper limb movement patterns, reduce compensatory movements and prevent secondary musculoskeletal damage (42). Wrist units are classified as passive, body-controlled and motorised devices, each with specific technical characteristics and clinical indications.

The clinical weight of these considerations is underscored by the interface and compensation literature discussed in Sections 5.2 and 5.7: by restoring even limited distal mobility, the wrist unit reduces the proximal compensatory movements that would otherwise load the shoulder girdle and trunk, so that its functional value extends beyond hand pre-positioning to the prevention of secondary musculoskeletal overload.

#### 4.2.1 Passive wrist units

Passive wrist units enable manual repositioning of the wrist using the opposite hand, without the need for an external power source. The addition of a passive wrist to a prosthesis with a single degree of freedom improves its functionality, demonstrating a positive impact on task performance (43). In a clinical study comparing flexible and static wrist units in eight transradial amputees with myoelectric prostheses, no significant differences were found in performance tests, but users reported greater satisfaction with the flexible wrists; the study concludes that individual needs, work requirements and prosthetic skills must be considered in the prescription (44).

State-of-the-art upper limb prostheses lack several degrees of freedom, forcing users to adopt compensatory movements; a passive articulated wrist with switchable dual stiffness, integrated into a body-controlled hydraulic prosthesis, has been shown to combine the advantages of rigid and compliant wrists by automatically selecting the appropriate level of stiffness (45).

#### 4.2.2 Powered wrist rotators

Powered wrist rotators represent a significant technological advancement, enabling active wrist movement through electric motors controlled by EMG signals or other sensory inputs. A two-degree-of-freedom prosthetic wrist, with two motors placed transversely within the hand shell and connected via a differential gear, is capable of producing wrist flexion/extension, pronation and supination, or a combination of both, creating a hand orientation that more closely matches grasping requirements (46).

An innovative approach to controlling motorised wrist rotators utilises inertial measurement units (IMUs): the controller detects upper arm abduction/adduction using a si*x*-axis IMU and uses this to control the wrist’s rotational speed, replacing the agonist/antagonist EMG signal and reserving it for hand control (47).

A comparative study between a biomechatronic prosthesis and a body-powered prosthesis involving 15 transradial amputees found greater user satisfaction with the biomechatronic prosthesis regarding supination/pronation, flexion/extension, pain and the ability to open a door; however, the ability to hold a cup and pick up an object was significantly better with the BP prosthesis (48).

### 4.3 Prosthetic elbow devices

Elbow prostheses are an important rehabilitation support for people with transhumeral amputations or elbow disarticulation. The prosthetic solutions available are divided into body-powered systems, externally controlled myoelectric systems, and hybrid systems that combine both technologies.

#### 4.3.1 Body-powered (BP) elbow units

BP elbow units utilise movements of the trunk and shoulder to operate a system of cables and a harness that transfers mechanical force to the prosthetic device. Their main advantages are their lightness, durability and the proprioceptive feedback provided by the cable, which helps users to sense the position of their elbow without the need for visual confirmation. Their main limitations are the wide range of shoulder movement required, which necessitates an adequate residual limb length and good shoulder mobility, and the inability to operate the elbow and the terminal device simultaneously without a complex commutation mechanism.

From a biomechanical perspective, a study specifically conducted on transhumeral amputees compared the forces, torques and pressures generated at the residual elbow during the use of three types of prosthesis (BP, myoelectric and air splint), revealing significantly different distributions compared to the natural limb and highlighting the importance of this information for selecting the most suitable device for the individual patient (49).

More recently, the application of additive manufacturing has opened up new possibilities for BP transhumeral prostheses: a 3D-printed device developed for an 8-year-old girl with a transhumeral malformation has been shown to be capable of performing elbow flexion/extension, shoulder rotation and forearm supination thanks to the patient’s shoulder movements, with a terminal grip force ranging from 85.3 N to 163.2 N (50).

Finally, a successful case of a BP prosthesis was reported in a patient who had undergone a transradial amputation following an explosion injury, where conversion to a transhumeral amputation with a minimally invasive humeral shortening osteotomy enabled the fitting of a body-powered prosthesis, used satisfactorily for daily activities, demonstrating excellent rotational control and ease of suspension at two years’ follow-up (51).

#### 4.3.2 Externally powered (myoelectric) elbow units

Externally controlled elbow units, known as myoelectric prostheses, use EMG signals captured from the residual muscles of the stump to control an electric motor integrated into the device. Myoelectric elbows offer significantly better performance than BP elbows in proximal amputees who lack sufficient shoulder mobility for cable-operated control, and provide a more natural appearance due to the absence of a harness.

The main challenge for transhumeral amputees lies in having to control several degrees of freedom (the motorised elbow, wrist and hand) with limited residual muscle groups, making current myoelectric control slow, sequential and unnatural (52).

One approach to overcoming these limitations involves utilising the mobility of the residual limb: by applying inter-joint coordination models derived from healthy subjects, it has been possible to synchronise the movement of the prosthetic elbow with the shoulder movement of the transhumeral patient, thus reducing compensatory trunk movements and achieving a more natural body posture compared to conventional myoelectric control (53).

Targeted Muscle Reinnervation (TMR) is a surgical strategy designed to increase the number of available control sites: in transhumeral patients, the technique involves redirecting residual nerves, including the musculocutaneous nerve for elbow control and the median nerve for hand control, to new muscle targets, with the aim of improving the quality and number of EMG signals for controlling the elbow, wrist and hand (54).

A study supports the creation of electroneuromuscular constructs using implanted electrodes and a bone-integrated interface: in a patient with a transhumeral amputation, this solution enabled the individual flexion and extension of all five fingers of the prosthetic hand, demonstrating a progressive increase in the amplitude of the myoelectric signal and an improvement in functional performance in activities representative of daily life (55). The comparative clinical efficacy, complication profile, and translational barriers of TMR and of the neural interface strategies introduced here are examined in detail in Section 5.6.

#### 4.3.3 Hybrid elbow–hand systems

Hybrid prostheses combine body-powered and myoelectric/externally powered components in a single device; they are primarily used for transhumeral amputations and shoulder disarticulations and most commonly include a body-powered elbow paired with a myoelectric terminal device (hook or hand). This configuration allows both components to operate simultaneously, providing the greater gripping force of the electric terminal device without placing a burden on the lightweight BP elbow unit. From a clinical and surgical perspective, prosthetists recommend the hybrid system featuring a body-powered elbow and an electric hand, as it improves operational efficiency by enabling faster positioning of the elbow and simultaneous control of the myoelectric hand (56).

Hybrid systems are particularly suitable for patients with proximal transhumeral amputations, for whom exclusively myoelectric control of all components would be excessively complex. Most transhumeral amputees opt for a BP elbow combined with a single-degree-of-freedom myoelectric hand and a myoelectric wrist for pronation–supination, as the sequential control of multiple joints via EMG signals is counter-intuitive and leads to the development of compensatory strategies involving the shoulder, back and contralateral limb (53).

One potential limitation is the discomfort caused by the harness (particularly in short transhumeral amputations, where shoulder muscle mass may be insufficient to generate adequate cable movement) and the fact that some users find the visible straps aesthetically unappealing.

## 5 Discussion

### 5.1 Comparative functional performance

Body-controlled prostheses have demonstrated advantages in terms of durability, training time, frequency of adjustments and feedback, although they still require improvements in control; myoelectric prostheses, on the other hand, improve aesthetics and reduce phantom limb pain, and are more readily accepted for low-intensity activities. However, current evidence is insufficient to conclude that either system offers a significant overall advantage: the choice of prosthesis should be based on the patient's individual needs (25).

This relative similarity also emerges in kinematic studies. Movements using myoelectric prostheses are slower and less fluid than those using body-controlled prostheses when handling objects, but these differences are not observed across all tasks, suggesting that no single type of prosthesis offers an absolute advantage in terms of movement quality (57).

With regard to transhumeral amputations, the simultaneous and proportional control of multiple degrees of freedom remains a challenge. Through the use of surgically implanted electroneuromuscular devices, it has been possible to achieve intuitive, simultaneous and proportional control of up to three degrees of freedom (hand, wrist and elbow) during prolonged home use, resulting in improved functional outcomes and reduced disability (58).

### 5.2 Biomechanical compensations and movement quality

An important aspect emerging from the current literature is that prosthetic performance should not be interpreted solely in terms of task completion, but also in relation to movement quality and the biomechanical compensations required to achieve the task. Several studies have shown that upper limb prosthesis users, particularly those with more proximal or mechanically constrained devices, often adopt altered motor strategies characterized by increased trunk displacement, shoulder recruitment, and reduced coordination between reaching and grasping components of movement (15, 16, 44). These compensatory patterns are especially evident when distal degrees of freedom are limited, such as in the absence of active wrist motion or in systems with reduced adaptability of the terminal device, forcing the user to rely on proximal segments to obtain adequate hand orientation and object alignment (39, 40, 59).

This matters clinically because compensatory movements can mask substantial inefficiencies in motor control. A prosthesis may appear effective on timed clinical tests, yet still rely on non-physiological strategies. These strategies increase mechanical load on the shoulder girdle and trunk, and may contribute to overuse and secondary musculoskeletal problems over time (60, 61). For this reason, the quality of movement should be considered a key outcome when evaluating upper limb prostheses, alongside speed and task success. From this perspective, the restoration of distal mobility (particularly at the wrist) and the development of more intuitive multi-joint control strategies appear essential not only for improving functional performance, but also for reducing compensatory burden and promoting more physiological movement patterns (14, 59).

### 5.3 Control limitations and the EMG signal problem

Despite the widespread adoption of myoelectric prostheses, the control of these devices remains one of the main challenges in upper limb prosthetics. Most commercially available systems rely on surface EMG signals acquired from residual muscles, which are used to generate control commands for the prosthetic components. Although this approach allows intuitive activation of specific movements, it is inherently limited by the variability and instability of the EMG signal.

The reliability of EMG-based control is affected by several physiological and technical factors that introduce variability in signal acquisition and interpretation. In particular, signal instability across sessions has been widely reported, with variations in electrode placement, skin impedance, and muscle condition leading to inconsistent control performance even in the same user over time (1, 62). Furthermore, external factors such as perspiration, socket fit, and electrode displacement can significantly alter signal quality during daily use, reducing robustness compared to controlled laboratory conditions (25). Muscle fatigue also contributes to signal variability, affecting both amplitude and pattern consistency and thereby influencing control accuracy during prolonged use (63).

Another critical issue is the so-called limb position effect, whereby changes in arm posture lead to variations in EMG signal patterns even when performing the same intended movement. This phenomenon complicates the generalization of control algorithms and reduces reliability in real-world scenarios (62, 63). In conventional control schemes, these limitations are often managed through sequential control strategies or mode switching, which require the user to alternate between different functions. While effective in enabling multiple degrees of freedom, this approach introduces an additional cognitive burden and can negatively impact task efficiency and intuitiveness, especially during complex activities involving multiple joints (14, 25).

More advanced control approaches, such as pattern recognition algorithms, aim to overcome some of these limitations by enabling the classification of multiple movement intentions based on EMG patterns. These systems have demonstrated improved performance and more natural control in experimental settings. However, their reliability remains strongly dependent on signal consistency and training conditions, and their robustness in daily life is still limited. Importantly, many advanced control strategies demonstrate promising results under laboratory conditions, where signal acquisition is optimized and environmental variability is minimized. However, their performance often degrades in real-world scenarios, where users are exposed to dynamic conditions, inconsistent electrode contact, and variations in muscle activation. This discrepancy highlights a critical gap between experimental performance and practical usability, representing one of the main barriers to widespread clinical adoption of advanced myoelectric control systems (64–66).

Overall, these findings indicate that improving the robustness, adaptability, and intuitiveness of EMG-based control remains a key priority in the development of upper limb prostheses. Addressing these challenges is essential not only for enhancing functional performance, but also for reducing cognitive load and facilitating long-term adoption in everyday life.

### 5.4 Sensory feedback: an unresolved challenge

One of the most relevant limitations of current upper limb prosthetic systems is the lack of effective sensory feedback, which represents a fundamental difference compared to the physiological functioning of the human limb. In natural conditions, motor control is tightly coupled with sensory input, including tactile and proprioceptive information, which are essential for modulating grip force, coordinating movement, and interacting efficiently with the environment. In contrast, most commercially available prostheses operate in an open-loop manner, forcing users to rely predominantly on visual feedback and cognitive strategies to control the device. This absence of intrinsic sensory information significantly affects the intuitiveness of control, increases cognitive load, and contributes to reduced embodiment and functional integration of the prosthesis (25, 66).

In recent years, considerable research efforts have been directed toward the development of sensory feedback systems aimed at restoring, at least partially, this missing information. Approaches such as sensory substitution through vibrotactile or electrotactile stimulation, as well as more advanced strategies involving direct neural interfaces, have shown promising results in improving object interaction, grip modulation, and user perception of the prosthetic limb (67). These systems can provide information related to contact events, force levels, or joint position, thereby enhancing the user's ability to perform functional tasks with greater precision and confidence.

However, despite these encouraging findings, the integration of sensory feedback into clinical practice remains limited. Many of the proposed solutions are still confined to experimental settings, often requiring complex hardware, invasive procedures, or extensive calibration and training.

Furthermore, issues related to long-term reliability, user comfort, and seamless integration with existing control systems have not yet been fully resolved. As a result, a substantial gap persists between technological potential and real-world implementation (66, 67).

### 5.5 Device abandonment: persistent challenges despite technological progress

The dropout rate for upper limb prostheses is the key indicator for assessing the true clinical impact of technological innovations. Historical data were already cause for concern: a systematic review covering the last 25 years has highlighted widespread consumer dissatisfaction despite technological advances, with dropout rates varying considerably depending on the type of device and the level of amputation (6).

Unfortunately, technological advances over the last decade do not appear to have substantially altered this trend; clinical practice still shows high rates of rejection of expensive devices, despite the academic solutions developed to address patients’ complaints (68).

Many advances in upper limb prosthetics have been primarily technology-driven, focusing on increasing degrees of freedom, improving mechanical complexity, or implementing advanced control algorithms, without always ensuring that these innovations translate into meaningful functional benefits for the user. Several studies have highlighted that user priorities often differ from engineering objectives, with greater emphasis placed on reliability, ease of use, comfort, and intuitive control rather than on maximal functionality or dexterity (6, 17). In this context, user-centered design has emerged as a critical paradigm in prosthetic development. Addressing this gap between engineering innovation and clinical applicability is essential for improving long-term acceptance and maximizing the functional impact of upper limb prosthetic systems (17).

According to a population-based study, the most frequently cited reasons for secondary non-use of prostheses are dissatisfaction with the comfort, function and control of the device; the risk of non-use is significantly higher for proximal amputations and among women (69).

In addition to technological factors, significant psychological and psychosocial factors also come into question; anxiety and depression are among the main contributors to prosthesis rejection, and high expectations regarding the technology are now a growing source of dissatisfaction (70). The level of amputation remains a key determining factor, especially in high or bilateral amputations, which constitute the most important predictor of the decision not to wear a prosthesis (71).

### 5.6 Surgical and neural interface strategies: from targeted muscle reinnervation to bidirectional and brain–computer interfaces

Three biological-interface strategies address the control limitations described above: TMR, implanted and osseointegrated neural interfaces, and brain–computer interfaces (BCIs), ordered by increasing invasiveness and decreasing clinical maturity.

TMR expands myoelectric control sites by redirecting residual nerves to spare muscles and simultaneously reduces post-amputation pain: a systematic review of 366 patients reported neuroma pain improvement in 75%–100% of cases and phantom limb pain improvement in 45%–80%, with numeric rating scale reductions of 2.4–6.2 points (72). The related regenerative peripheral nerve interface (RPNI) further amplifies and segregates EMG signals for pattern-recognition control (73).

These benefits come at a surgical cost: complication rates of 13%–31% have been reported, predominantly delayed wound healing (72, 74), and the evidence base remains dominated by retrospective series rather than controlled trials.

Osseointegrated neural interfaces couple intramuscular electrodes with skeletal anchoring to create a stable bidirectional link: in one transhumeral case, a year of home use yielded 26% improvement in control performance and a 34.7% reduction in required EMG signal magnitude, consistent with genuine motor learning (75). Electrode comparisons between intramuscular and epimysial types show greater channel isolation with the former but no significant advantage in signal separability, suggesting muscle-specific selection is preferable (76); psychosocial benefits including improved self-image and social participation have also been documented (77).

BCIs remain the least mature option: multimodal fusion of cortical and muscular signals is increasingly favoured (78), but non-invasive approaches are limited by low signal-to-noise ratios and invasive ones by uncertain long-term stability, keeping BCI-driven prosthetic control predominantly experimental.

Across all three strategies, the key barriers to translation are not conceptual but practical: surgical invasiveness, out-of-laboratory reliability, absence of reimbursement pathways, and difficulty scaling prototype results, reinforcing that technological sophistication delivers clinical value only when matched to real-world robustness and to the priorities of the individual user.

### 5.7 The residual limb–socket interface: materials, fit, and wearing comfort

The preceding sections have considered prosthetic devices largely in terms of their distal mechanics and control. Yet the determinant most consistently associated with sustained prosthesis use lies more proximally, at the interface between the residual limb and the socket. A comfortable, well-fitting socket has been described as the single key factor governing how long (or indeed whether) a person with an upper limb amputation can tolerate wearing a prosthesis (79). This observation reframes socket design not as an ancillary fitting detail but as a primary determinant of clinical success, on a par with the control interface.

The interface poses a structural tension that is particularly acute in myoelectric systems. The socket must remain comfortable throughout a full day of wear while simultaneously maintaining stable contact between the control electrodes and the underlying muscles; comfort and control dissatisfaction are together among the most frequently cited contributors to device abandonment (80). Rigid sockets that secure reliable electrode contact may compromise comfort, whereas designs optimised for comfort may permit slippage and inconsistent signal acquisition. A direct comparison of a conventional transhumeral socket with a compression-released stabilised design illustrated this trade-off precisely: the stabilised socket afforded better control of the residual limb and less slippage, yet users reported lower comfort and required additional fitting visits (79). Adjustable solutions, in which electrode compression can be tuned by the user, have been proposed to reconcile these competing demands, with encouraging early results (80).

Materials selection contributes a further dimension. Passive and cosmetic devices rely on silicone and thermoplastic elastomers chosen for skin-tone matching and low maintenance, whereas additively manufactured devices depend on thermoplastics whose mechanical properties bear directly on durability and grip strength. The introduction of more advanced printable materials may mitigate these limitations, but tends to erode the low cost and wide accessibility that constitute the principal advantage of the 3D-printed approach (38). Across materials and socket designs alike, the recurring lesson is that real-world performance is constrained less by the sophistication of the terminal device than by the quality, comfort, and stability of its coupling to the body, a consideration that aligns directly with the user-centred priorities emphasised throughout this review.

## 6 Limitations

The evidence base for upper limb prosthetics is constrained by intrinsic and persistent methodological limitations, several of which bear directly on the interpretation of this review.

First, the primary studies in this field consist predominantly of uncontrolled cross-sectional analyses and small case series (frequently *n* < 30), reflecting the low prevalence of upper limb amputation and the marked heterogeneity of the amputee population. Randomised controlled trials remain rare, and the few available have compared prosthetic management strategies or device types almost exclusively in transradial amputees, leaving more proximal presentations (transhumeral and shoulder-level amputation) and bilateral amputation without high-level comparative evidence. This review therefore reflects, rather than overcomes, the current evidentiary ceiling of the field.

Second, outcome measurement is markedly heterogeneous across studies. Functional performance is assessed with a range of instruments, among them the Southampton Hand Assessment Procedure (SHAP), the Box and Block Test (BBT), the Jebsen–Taylor Hand Function Test, the Disabilities of the Arm, Shoulder and Hand (DASH) questionnaire, and the Assessment of Capacity for Myoelectric Control (ACMC), which capture distinct constructs and are not directly interconvertible. Critically, the minimal clinically important difference (MCID) for these instruments has not been characterised for upper limb prosthesis users (81). This absence is not merely a constraint on the present synthesis but a field-level gap: it precludes meaningful cross-study comparison and quantitative pooling. The present review makes that gap explicit and argues that the standardisation of outcome measures, together with the derivation of population-specific MCID values, is a precondition for the very quantitative evidence synthesis that the field currently cannot support.

Third, consistent with its deliberately narrative design, this review did not follow a formal systematic-review methodology: no standardised risk-of-bias assessment was performed, and no quantitative synthesis or meta-analysis was undertaken. This choice was dictated by the heterogeneity of study designs and outcome measures described above, which renders the assumptions underlying meta-analytic pooling untenable in this field. The findings should accordingly be read as a qualitative, integrative overview of the current state of the art rather than as a quantitative comparison of device performance or abandonment rates. Article selection was guided by topical relevance, which may introduce a degree of selection bias; the restriction to English-language sources may additionally introduce language bias and under-represent regionally significant work published in other languages.

Fourth, most studies have enrolled predominantly male participants, limiting generalisability to women and children. The gap between controlled laboratory performance and real-world prosthetic use remains substantial (82) and has only recently begun to be characterised through wearable sensors, head-mounted cameras, and on-device usage logging. Many studies emphasise short-term performance and under-report long-term adaptation, user learning, fatigue, and sustained device use, so that important determinants of real-world acceptance may be under-represented in the current literature.

Finally, the scope of this review was intentionally limited to the technological and functional dimensions of hand, wrist, and elbow devices. Other important dimensions, including rehabilitation pathways, motor (re)learning, training protocols, and the operationalisation of clinical decision-support tools for individualised prescription, were not examined in detail and represent dedicated objects for future work.

## 7 Conclusions

This narrative review examined the landscape of upper limb prostheses in relation to three main levels of amputation: hand, wrist and elbow, summarising the evidence regarding structure, control mechanisms, functional outcomes and user satisfaction (Table 1). The overall view that emerges is one of significant technological progress accompanied by persistent clinical and user-centred challenges, which have not been fully resolved despite decades of research and development.

Across the entire spectrum of hand prostheses, from passive and cosmetic prostheses to BP systems, and from single-DOF and multi-jointed myoelectric hands to 3D-printed alternatives, no category demonstrated across-the-board superiority. Passive prostheses, although functionally limited, meet the psychosocial needs of a substantial proportion of users, particularly those with congenital limb agenesis. BP devices remain competitive due to their durability, low weight, intrinsic proprioceptive feedback and reliability in challenging environments. Multi-jointed myoelectric hands represent the most technologically advanced commercial option. 3D printing has introduced significant opportunities for customisation and cost reduction, particularly in the paediatric population and in resource-limited settings, although limitations in mechanical durability and fine motor precision restrict their wider clinical adoption.

Wrist prostheses have historically received insufficient attention relative to their functional importance. Evidence suggests that restoring even limited wrist mobility, whether through passive, body-controlled or motorised rotators, reduces compensatory movements in the shoulder and trunk, prevents secondary musculoskeletal overload and improves overall performance. At the elbow, the challenge of simultaneously and intuitively controlling multiple DOF, using a limited number of residual muscle groups, remains the central unresolved issue in transhumeral prosthetic rehabilitation. BP elbow units offer tactile feedback and mechanical reliability, but require adequate residual limb length and shoulder mobility. Myoelectric elbow prostheses offer a more natural appearance and are better suited to patients with proximal amputation and limited shoulder mobility; however, sequential control paradigms remain cognitively demanding and prone to compensatory strategies. Hybrid configurations, which combine a body-driven elbow with a myoelectric end-effector, represent a pragmatic clinical compromise that leverages the complementary strengths of both technologies.

**Figure 1:**
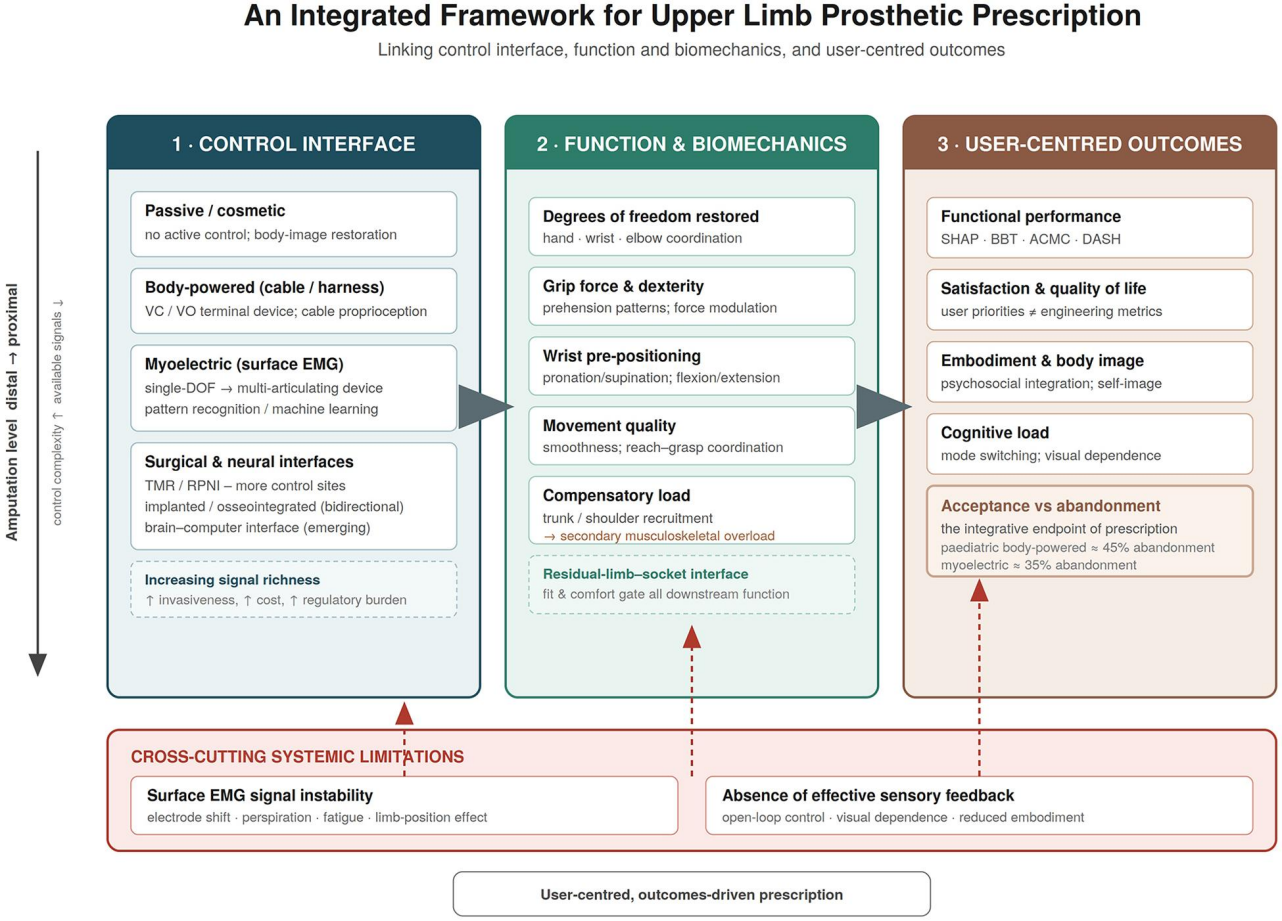
An integrated framework for upper limb prosthetic prescription. The framework links three domains: the control interface (passive, BP, myoelectric, and surgical/neural strategies, ordered by increasing signal richness and invasiveness); function and biomechanics (degrees of freedom restored, grip and dexterity, wrist pre-positioning, movement quality, and compensatory load, gated by the residual limb–socket interface); and user-centred outcomes (functional performance, satisfaction and quality of life, embodiment, cognitive load, and ultimately acceptance versus abandonment). Two cross-cutting systemic limitations, the instability of the surface electromyographic signal and the absence of effective sensory feedback, constrain the flow across all three domains. The dashed feedback loop denotes user-centred, outcomes-driven prescription and design, in which real-world outcomes inform device selection and development. The vertical axis indicates that more proximal amputation levels entail greater control complexity and fewer available control signals.

Two systemic limitations apply across all categories of prostheses and levels of amputation. First, surface EMG, still the dominant control interface, remains vulnerable to electrode displacement, perspiration, fatigue, and inter-session variability; pattern-recognition algorithms show considerable laboratory potential, but their real-world reliability remains an active area of research. Second, the lack of sensory feedback keeps users dependent on vision, adding a cognitive burden that is repeatedly implicated in device abandonment (Figure 1).

Surgical and neural interface strategies (from TMR to osseointegrated bidirectional interfaces and, prospectively, BCIs) offer progressively richer control at the cost of increasing invasiveness, and their translation will depend less on conceptual novelty than on demonstrating real-world reliability, cost-effectiveness, and reproducibility beyond single-case prototypes. Looking forward, the field stands at a crucial crossroads. Advances in machine learning and bidirectional sensory feedback offer significant promise for bridging the gap between the performance of prosthetic limbs and that of biological limbs. Fully realising this potential will require not only technological progress, but also a paradigm shift in how devices are evaluated and developed: from laboratory-centred, technology-driven approaches to user-centred, outcomes-driven models that systematically integrate real-world usage data, patient-reported outcomes and long-term follow-up. Only through this integrated and person-centred approach, combining intuitive control, meaningful sensory restoration, and real-world validation, will the full potential of prosthetic technology translate into significant improvements in the daily lives of people with upper limb amputations.
